# Experiences in the Antiseptic Treatment of Typhoid Fever1Read before the Medical Association of Central New York, at Rochester, October 20, 1896.

**Published:** 1897-03

**Authors:** Charles H. Richmond

**Affiliations:** Livonia Station, N. Y.


### EXPERIENCES IN THE ANTISEPTIC TREATMENT OF TYPHOID FEVER.[1]

By CHARLES H. RICHMOND, M. D., Livonia Station, N. Y.

WHEN the antiseptic treatment of typhoid fever was first introduced I was somewhat sceptical as to its practicability, owing to the distance such a remedy would have to travel before reaching the point of systemic infection, it being thereby likely to lose much of its power. Moreover, the delay which usually elapses before a physician is called and before an accurate diagnosis is made, enables the disease to advance sufficiently for the development of changes in the intestinal tract which will require time to repair.

I have treated typhoid fever for many years antipyretically, according to Liebermeister's plan, with baths and quinine, cases of all grades, without the loss of a single case, but after the discovery of the coal tar preparations, so much had been said about the value of acetanalid that I applied it in two or three cases. One of these died at the end of about eight weeks, of general dropsy and general breaking down of the system, the remedy seeming to cause a disintegration of the blood. I did not treat many in that way.

Disliking to be out of fashion, I employed the sulpho-carbolate of zinc treatment in three or four cases. Aside from the remedy causing irritation of the stomach, it did not seem to cut short the disease in any case, although it may have mitigated the force of it somewhat. My first or second case so treated died of intestinal hemorrhage during the fourth week.

I used tincture of iodine and carbolic acid, with a ten-grain dose of calomel every four days, in a number of cases ; I do not remember how many. The cases all recovered, with an average duration of about three weeks, with the temperature somewhat modified by the remedy. The patients were rather weak at the termination of the fever, but they recovered without any very bad symptoms supervening.

During the past three years I have treated all my cases with salol, supplemented with a sponging of the surface. I will mention especially the results in my cases of the present summer and autumn—nine in all. In one the duration was twenty-one days from

---

1. Read before the Medical Association of Central New York, at Rochester, October 20, 1896.

the institution of the treatment; in one, fourteen days ; in five, ten days and in two seven days. In several of the cases the afternoon temperature reached 104, but did not remain at that point more than two or three days. Those of the longest duration were not seen until the patients had been ill for several days. One of the cases which lasted ten days seemed about to terminate at the end of a week, when the treatment was discontinued by a misunderstanding, and the fever returned, only terminating at the end of the tenth day. In another of the ten-day cases, at the termination of the fever proper, a circumscribed pneumonia took place, attended with fever lasting about four days. In all the cases treated by salol during the three years, only three have apparently run a full course of three or four weeks, and in these the temperature had been reduced from an afternoon registration of 104 to 102 or below during the remainder of the course, while the patients at the termination were usually in good condition and gained rapidly.

The reason of the efficacy of salol over other antiseptics in typhoid fever is, I suppose, that it only breaks up into its component parts after having passed well down in the smaller intestines, allowing the antiseptic elements to come in contact with the affected parts. If ulceration has taken place before the treatment is begun, while the symptoms may be considerably mitigated, it will be impossible to arrest the case until the process of repair can take place ; hence, the earlier the treatment the more certainty of arresting the course of the disease. I usually use the drug in seven-grain doses in adults, every three hours, for the first few days, after which, if the patient does well, the dose is reduced to five grains. If the morning temperature drops in a marked degree, or if profuse diaphoresis occurs, two or three of the early morning doses are omitted. I always direct care in its administration after midnight, and to omit if the urine becomes black. One point it will be well to bear in mind—salol should be always given in the crystal form and never in pill, if certainty of effect is desired.

So far as other treatment is concerned, plenty of fresh air, water and liquid nourishment are required, and I have never seen harm result from the bath, only I never use it very cold. A bath is much more effective in reducing temperature than sponging, but sponging will answer very well when the symptoms are not urgent.

